# Dengue seroprevalence and force of primary infection in a representative population of urban dwelling Indonesian children

**DOI:** 10.1371/journal.pntd.0005621

**Published:** 2017-06-15

**Authors:** Ari Prayitno, Anne-Frieda Taurel, Joshua Nealon, Hindra Irawan Satari, Mulya Rahma Karyanti, Rini Sekartini, Soedjatmiko Soedjatmiko, Hartono Gunardi, Bernie Endyarni Medise, R. Tedjo Sasmono, James Mark Simmerman, Alain Bouckenooghe, Sri Rezeki Hadinegoro

**Affiliations:** 1 Department of Child Health, Faculty of Medicine Universitas Indonesia, Jakarta, Indonesia; 2 Epidemiology and Health Economics & Outcomes Research, Sanofi Pasteur Asia & JPAC Region, Singapore; 3 Eijkman Institute for Molecular Biology, Jakarta, Indonesia; 4 Medical Affairs & Clinical Sciences Department, Sanofi Pasteur Asia and JPAC Region, Singapore; Universidad de Buenos Aires, ARGENTINA

## Abstract

**Background:**

Indonesia reports the second highest dengue disease burden in the world; these data are from passive surveillance reports and are likely to be significant underestimates. Age-stratified seroprevalence data are relatively unbiased indicators of past exposure and allow understanding of transmission dynamics.

**Methodology/Principal Findings:**

To better understand dengue infection history and associated risk factors in Indonesia, a representative population-based cross-sectional dengue seroprevalence study was conducted in 1–18-year-old urban children. From October to November 2014, 3,210 children were enrolled from 30 geographically dispersed clusters. Serum samples were tested for anti-dengue IgG antibodies by indirect ELISA. A questionnaire investigated associations between dengue serologic status and household socio-demographic and behavioural factors. Overall, 3,194 samples were tested, giving an adjusted national seroprevalence in this urban population of 69.4% [95% CI: 64.4–74.3] (33.8% [95% CI: 26.4–41.2] in the 1–4-year-olds, 65.4% [95% CI: 69.1–71.7] in the 5–9-year-olds, 83.1% [95% CI: 77.1–89.0] in the 10–14-year-olds, and 89.0% [95% CI: 83.9–94.1] in the 15–18-year–olds). The median age of seroconversion estimated through a linear model was 4.8 years. Using a catalytic model and considering a constant force of infection we estimated 13.1% of children experience a primary infection per year. Through a hierarchical logistic multivariate model, the subject’s age group (1–4 vs 5–9 OR = 4.25; 1–4 vs. 10–14 OR = 12.60; and 1–4 vs 15–18 OR = 21.87; p<0.0001) and the number of cases diagnosed in the household since the subject was born (p = 0.0004) remained associated with dengue serological status.

**Conclusions/Significance:**

This is the first dengue seroprevalence study in Indonesia that is targeting a representative sample of the urban paediatric population. This study revealed that more than 80% of children aged 10 years or over have experienced dengue infection at least once. Prospective incidence studies would likely reveal dengue burdens far in excess of reported incidence rates.

## Author summary

Indonesia reported to the WHO the world’s second highest average number of dengue cases and the highest in Asia from 2004 to 2010. These passive surveillance reports vary widely within the country and are likely to be a severe under-estimation of the full disease burden as frequently only dengue haemorrhagic fever is captured. Understanding the intensity of dengue virus transmission and associated risk factors nationwide is necessary to guide and prioritize appropriate prevention and control measures against dengue disease, especially considering the availability of the first dengue vaccine and recent recommendations for its use in areas of high endemicity, as measured by seroprevalence and other indicators. Age-stratified seroprevalence data provide robust estimates of past exposure and can inform on transmission intensity. Therefore, we conducted a seroprevalence study of anti-dengue IgG antibodies in a representative sample of urban-dwelling Indonesian children. We found an overall dengue seroprevalence of 69.4% with half of the children having been infected at least once by the age of 5 years. Age of the subject and the number of dengue cases diagnosed in the household were associated with serostatus. These results confirm the high dengue disease burden in Indonesia and the urgency of implementation of effective prevention and control measures.

## Introduction

Dengue is an arbovirus transmitted to humans via the bites of infected *Aedes* mosquitoes. It is the most rapidly spreading mosquito-borne viral disease with a global incidence that has increased 30-fold over the last 50 years [1]. While reliable burden estimates remain elusive, two studies have estimated the global symptomatic disease burden to be 96 million and 58.4 million cases/year, with 70–80% of cases occurring in the Asia-Pacific region [2, 3]. Traditionally an urban disease, dengue disease is increasingly reported in rural areas and its geographic range has expanded to more than 125 tropical countries [1]. There is no specific antiviral treatment; clinical management is focused on careful fluid management and detection of early warning signs of severe disease. Historically, prevention measures have focused on vector control, education and behavioural changes to reduce interactions between humans and vector mosquitoes [4, 5]. Improved clinical management and public awareness have contributed to declining case fatality rates to below 1% in most countries [1]. While this represents important progress, overall dengue incidence continues to rise and fatalities remain unacceptably high, suggesting that traditional control approaches are not sufficient. Vector control measures are important yet operationally challenging, of variable effectiveness and costly to sustain [6]. Routine vaccination is becoming a reality: several dengue vaccines are at different stages of clinical development [7] and a chimeric tetravalent vaccine from Sanofi Pasteur is being licensed in an increasing number of countries in Latin America and Asia [7, 8]. In this new era of dengue as a vaccine-preventable disease, an accurate understanding of disease burden and transmission patterns will be essential to inform vaccine policy decisions.

Dengue is hyper-endemic with frequent epidemic cycles in Indonesia. The disease is most common in urban areas and in recent years has reportedly spread to smaller, more rural villages. Reported incidence remains highest in children 1–15 years of age, but since the 1980s incidence in persons over 15 years of age has gradually increased [9, 10]. Reporting of dengue haemorrhagic fever (DHF) is mandatory in Indonesia and the country typically reports the highest number of cases in the WHO Southeast Asia Region [1]. Between 2001 and 2011, there was an average of 94,564 reported cases and between 472 and 1,446 reported deaths per year [1, 11]. Dengue disease reporting is acknowledged by Indonesian experts to be incomplete and to vary widely between provinces, with reported incidence rates ranging from 2.2 to 168.5 cases per 100,000 inhabitants in 2013 [12].

An improved understanding of dengue epidemiology, burden and its dynamic characteristics are important for public health planning. Seroprevalence studies in healthy volunteers provide information on infection history in the population, from which inferences about disease burden may be drawn. Since age reflects duration of exposure, age-stratified data provide insights into transmission dynamics [13–17]. There is a lack of dengue seroepidemiological data from Indonesia and no previous study has used a population representative sample of urban Indonesian children [18–20]. This is a particularly important gap as it will provide information on whether the variations in reported incidence from different Indonesian provinces are reflective of underlying transmission dynamics or to the result of the reporting or surveillance practices employed. We conducted a seroprevalence study in urban-dwelling Indonesian children to improve understanding of dengue epidemiology and infection risk factors and inform future dengue vaccine policy decisions.

## Methods

The present study is reported according to STrengthening the Reporting of OBservational studies in Epidemiology (STROBE) recommendations (supporting information file).

### Ethic statement

The protocol was reviewed and ethical approval was obtained from the Health Research Ethics Committee of Faculty of Medicine of University of Indonesia.

### Study area

Indonesia is the largest country in Southeast Asia, with an area of 1.91 million km^2^. The country has a population of 252.2 million living on five main islands and four archipelagos (>17,000 islands) administratively divided into 34 provinces [21]. In 2014/2015, approximately 60% of Indonesians were living on the island of Java and 53.3% lived in urban areas [21, 22]. Indonesia is divided into five administrative levels: provinces (n = 34), regencies (n = 416), cities (n = 98), subdistricts (n = 7,024), and villages (n = 81,626). Villages are considered either as rural (*desa*) or urban (*kelurahan*) based on population density, percentage of agricultural household and number of urban facilities such as schools and hospitals [21, 23].

### Sampling design

A population-based cross-sectional study design was adapted from the World Health Organization (WHO) Expanded Program on Immunization (EPI) cluster survey method. This approach considers 30 clusters as an adequate number for their means to be normally distributed, thus permitting statistical theory based on the normal distribution to be used to analyse the data [24, 25]. Based on the probability proportional to population size, 30 urban subdistricts were selected using demographic data from 2009 or 2010, provided by the Sub-Directorate of Statistical Services and Promotion, Statistics Indonesia.

**Fig 1:**
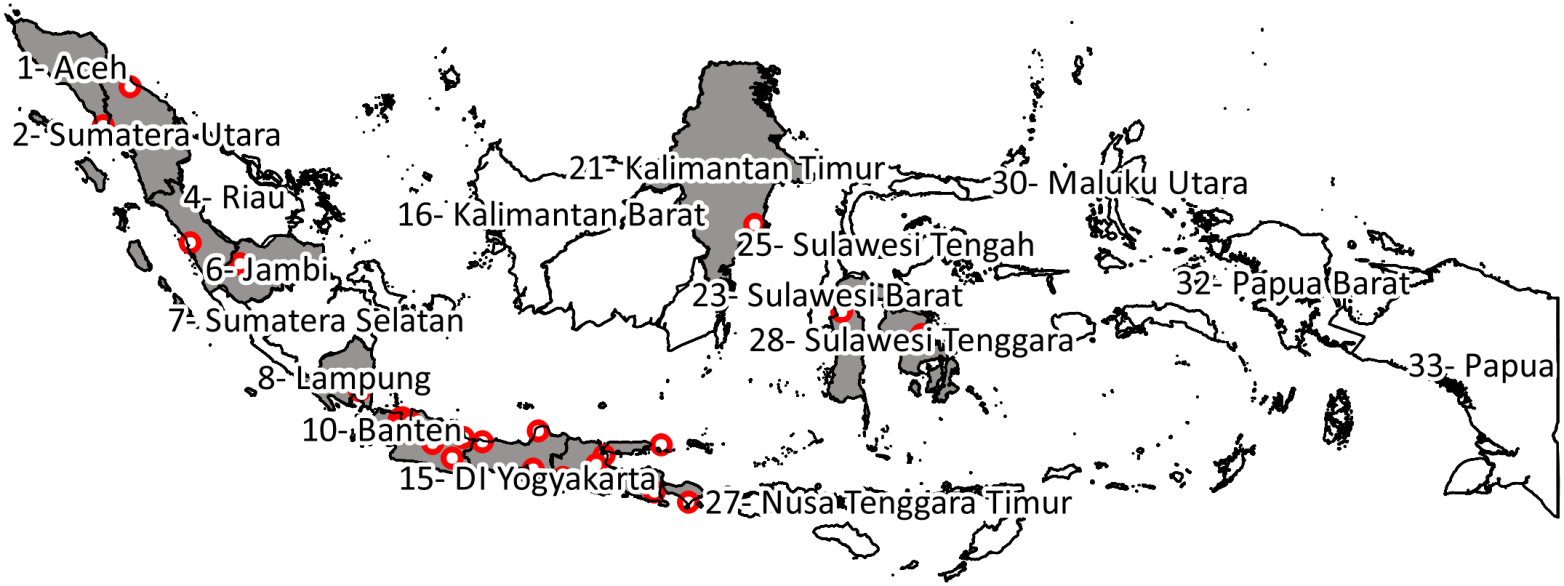
Map of Indonesia showing the ranking of provinces from West to East and study sites’ geographic distribution. Provinces with at least one site are coloured in grey. Developed with QGIS 1.8.0 using data from Global Rural-Urban Mapping Project^18^.

The geographical coordinates of Indonesian administrative units were retrieved from the *Global Rural-Urban Mapping Project*, maintained by the *Socioeconomic Data and Applications Center* [26]. Provinces were listed based on their mean geographical coordinates from West to East (Fig 1) and the cumulative urban population of their subdistricts was calculated using 2010 population data. To ensure the population of clusters was sufficient to enrol the desired sample, a minimum population of 1,000 persons per subdistrict was defined and any smaller subdistricts were removed from the list. The first cluster was selected by generating a random number between 1 and 1/30^th^ of the total urban population, using Epi Info Version 7, and selecting the first subdistrict for which the cumulative population was superior or equal to this random number. Subsequent clusters were selected by adding 1/30^th^ of the urban population to the random number and selecting the first corresponding subdistrict for which cumulative population was higher or equal so that:

$${\text{Cluster}}_{\text{i}}\text{ cumulative population }\geq \text{ random number }+\text{ i}*1/30\text{ of urban population}$$

The 30 subdistricts selected by this method are listed in Appendix 1. Each subdistrict in Indonesia contains one main health centre (*puskesmas kecamatan*) whose catchment area was the site of the study. Households in the five neighbourhood associations located closest to the health centre (each comprising 30–50 households, giving a total of 150–250 households) were eligible to participate in the study. Household visits were conducted, inviting one child from each household to participate, until the sample size was reached. A table indicating the required number of children from each of four age groups was provided to the health centre study teams. If a household had only one eligible child, the child was invited. When a household had several eligible children, a child in the age group with the fewest children already participating was selected. Towards the end of the survey, survey teams were allocated a specific number of subjects in each age group to recruit to avoid over-sampling. If the parents refused the participation of the selected child, the household was not included. This process was continued until the desired sample size was achieved in each of the 30 clusters.

### Sample size

The sample size was calculated using EpiInfo Version 7 to estimate seroprevalence in each of four age groups (1–4, 5–9, 10–14 and 15–18 years old) with 95% confidence, a margin error of 5% and accounting for clustering with a design effect of 2. The expected national seroprevalence, based on Indonesian expert opinion and published regional data [14, 19, 27, 28], was 25% in the 1–4-year-old group, 45% in the 5–9-year-old group, 55% in the 10–14-year-old group and 65% in the 15–18-year-old group. To account for incomplete data, a 10% contingency was applied. The total sample size was 3,210 children, 660 from the 1–4-year-old group (22 per cluster), 870 from the 5–9-year-old group (29 per cluster), 870 from the 10–14-year-old group (29 per cluster) and 810 from the 15–18-year-old group (27 per cluster). In total, 107 children were enrolled in each cluster.

### Enrolment

The study was presented to families during monthly neighbourhood association meetings. After household visits, eligible subjects were invited to the healthcare centre for enrolment and blood sampling if they were healthy, 1–18 years of age on inclusion day, and had lived in the location for at least 1 year. An informed consent form was signed by a parent or legal guardian, and by the subject if aged 13–18 years. Subjects aged 8–12 years provided signed assent.

A questionnaire was administered to collect information on demographics, knowledge of dengue symptoms and transmission, vector control practice, and medical history in the household.

### Blood sampling and laboratory analysis

For each subject, 2mL of venous blood was drawn into plain vacutainer tubes. After centrifugation, serum aliquots were frozen at -20°C before refrigerated transport by courier to a central laboratory for analysis. Each specimen was tested for dengue IgG antibodies by ELISA using the commercial Panbio Dengue IgG Indirect ELISA kit (sensitivity = 96.3%; specificity = 91.4–100% according to manufacturer’s instructions; Panbio, Alere, Australia) [29]. Samples were considered positive for previous dengue infection according to the standard protocols of the manufacturer (Panbio units <9 is negative; 9–11 is equivocal; and >11 is positive).

### Data analysis and statistics

All analyses were run using SAS 9.4.

#### Dengue antibody seroprevalence and associations between serologic status and socio-demographic and behavioural factors

The statistical unit was the individual subject.

Seroprevalence and the 95% confidence interval (95% CI) were calculated taking account of the cluster effect. Univariate logistic regression was used to identify variables significantly associated with serologic status. As the data structure was hierarchical with subjects included in clusters, hierarchical logistic regression models were used to consider subject intra-cluster correlation. The clusters account for the random effect and the covariates were taken as fixed effects. As these analyses were considered exploratory, a level of significance (p-value) of <0.15 was applied at univariate level.

The multivariate hierarchical model was reduced by applying a backward descending selection of the non-significant variables at p-value >0.05.

The final model was:

$$P\;\left(Y=\frac{1}{0}\right)ij=\beta \;\times \;Parameter\;+\;\mu \;\times \;cluster\;+\;\varepsilon$$

Where $P\;\left(Y=\frac{1}{0}\right)ij$ was the probability for a *j* subject from a *i* cluster to be seropositive, the *β*s were the fixed effect describing the subject variables associated with socio-demographic and behavioural factors, *μ* the cluster random effect and *ɛ* the error term.

#### Median age of conversion

The median age of seroconversion was estimated by fitting a weighted linear regression model to age-specific seroprevalence data. Seroprevalence data were transformed into probits and age values were log transformed to fit the model [30, 31]. However, goodness of fit parameters were not respected. Therefore, a simple linear regression was used.

#### Force of infection

Catalytic models use seroprevalence data as cumulative markers of past infections that result in life-long immunity from which force of primary infection estimates can be derived. [32, 33], Two force of infection models were developed to describe the rate of infection over the last 18 years and to examine its variability over time. The first model assumed a constant force of infection (model 1) and the second one assumed a force of infection that varied with age (model 2) [13].

The probability of a person living in the area being infected in one year, the force of infection, is estimated by [34]:

$$-\;p\;=\;1-e^{-\mu }$$

Where μ is the mean number of infections per year.

The variable force of infection model can be estimated by allowing a separate risk of infection for each age group, were p*i* is the mean number of infections per year for the *i*^th^ age group and A is the age midpoint of the *i*^th^ age group [34]. By fitting a binomial model with a complementary log-log link function and by using X = log(A) as an offset term, α = log(μ) can be estimated as an intercept parameter [34]. The probability of being infected for the *i*th group at midpoint age A is p*i* = 1- exp(-μ*i* A*i*), so that:

Log(−log(1−pi)) = log(μi) + log(Ai)

## Results

### Site selection and baseline demographics

**Fig 2:**
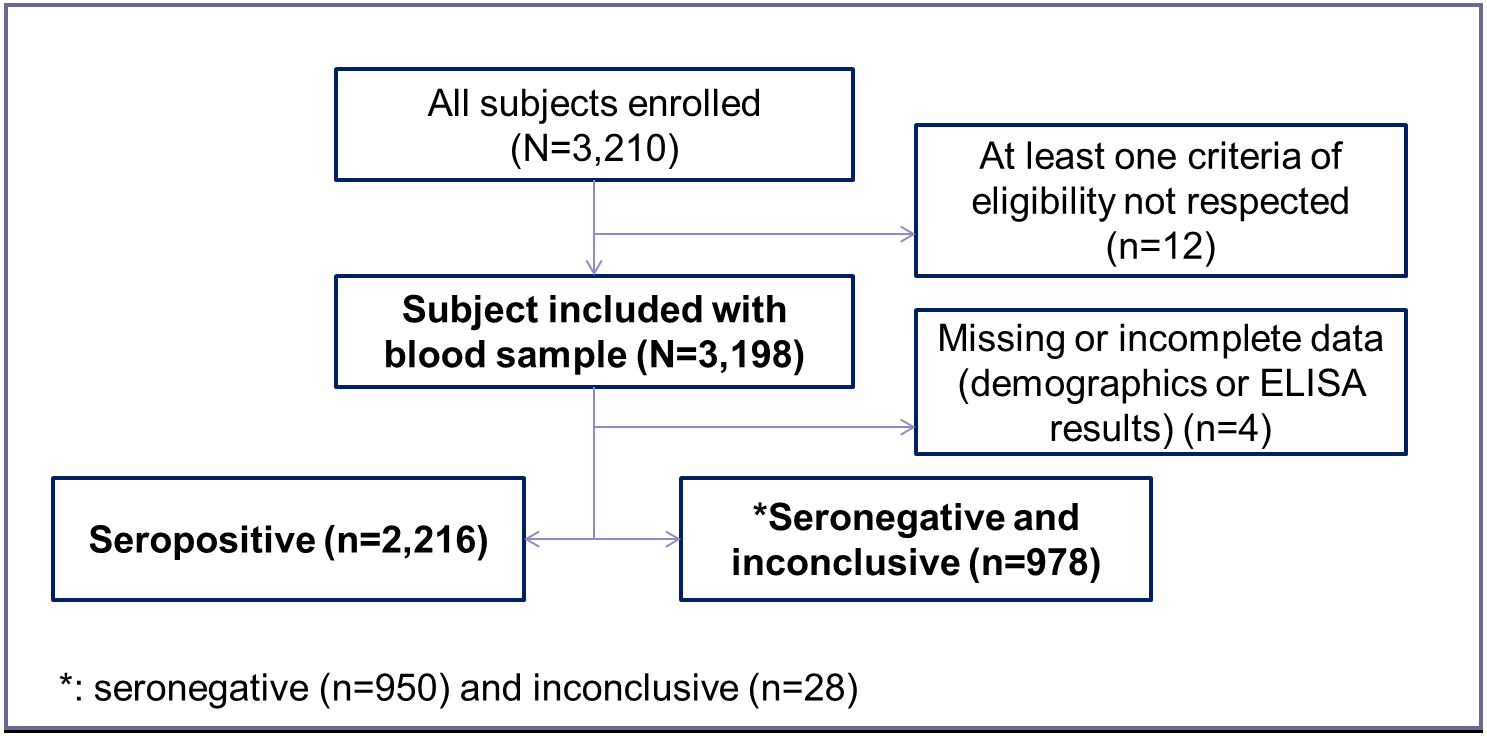
Study flow chart.

From a total of 6,299 Indonesian subdistricts, 2,823 with urban population were identified, 2,756 of which had an urban population >1,000 and were thus used for sampling. A map of the 30 selected clusters is presented in Fig 1. From 30 October 2014 to 27 November 2014, a total of 3,210 subjects were enrolled in the study; 39 subjects (1.2%) were excluded due to at least one criteria of eligibility not being fulfilled and four subjects (0.1%) due to missing or incomplete data (demographic or serologic status result). A total of 3,194 subjects (98.7%) were included in the analyses (Fig 2); there were 107 subjects per site with the exception of four sites with 106 subjects, three sites with 105 subjects and one site with 101 subjects.

There were 672 subjects in the 1–4-year-old age group, 861 subjects in the 5–9-year-old age group, 886 in the 10–14-year-old age group and 775 in the 15–18-year-old age group. Among them, 47.8% were male and the mean age was 9.7 years.

### Dengue antibody seroprevalence and association between serologic status and socio-demographic and behavioural factors

**Fig 3:**
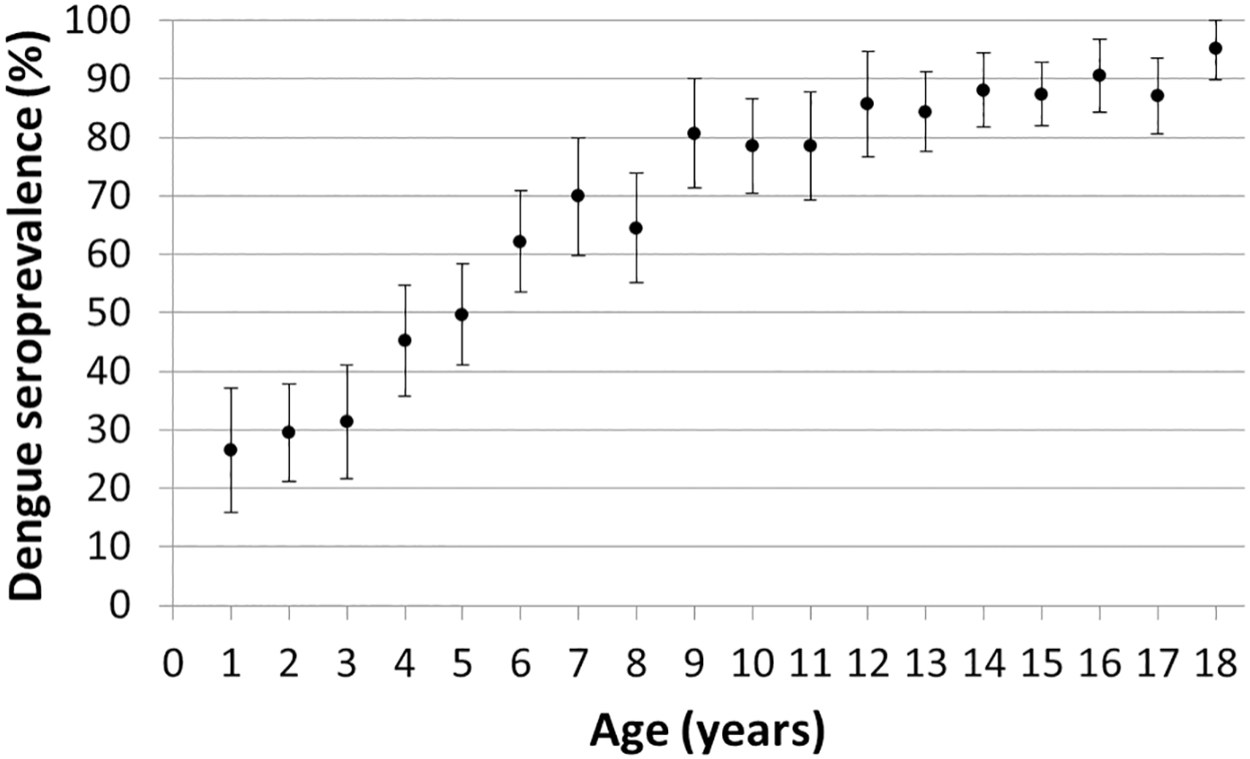
Mean age-specific dengue antibody seroprevalence distribution and 95% confidence interval.

The age-specific seroprevalence ranged from 26.4% (95% CI: 15.8–37.1) in those aged 1-year-old to 95.3% (95% CI: 89.8–100) in the 18-year-old subjects (Fig 3). The median age at seroconversion was 4.8 years. The overall nationwide seroprevalence was 69.4%, with a minimum of 34.6% and a maximum of 87.9% observed per site, and the seroprevalence per age group was 33.8% in the 1–4year-old group, 65.4% in the 5–9-year-old group, 83.1% in the 10–14-year-old group and 89.0% in the 15–18-year-old group (Table 1).

**Table 1:** Description of subject demographics and knowledge of dengue symptoms and transmission, vector control practice, and medical history in the household and results of the univariate hierarchical logistic model.

Variable	Number samples (%)	Response rate	Seroprevalence [95% CI]	Odd ratio [95% CI]	P-value
**Overall urban seroprevalence**	3,194		69.4 [64.4–74.3]		
***Subject demographics***					
**Gender**		100%			
Male	1527 (47.8%)		67.4 [62.4–72.5]	Ref.	0.018
Female	1667 (52.2%)		71.1 [65.9–76.3]	1.21 [1.03;1.41]	
**Age**		100%			
1–4	672 (21.0%)		33.8 [26.4–41.2]	Ref.	<0.0001
5–9	861 (27.0%)		65.4 [59.1–71.7]	4.40 [3.50;5.52]	
10–14	886 (27.7%)		83.1 [77.1–89.0]	12.95 [10.0;16.79]	
15–18	775 (24.3%)		89.0 [84.0–94.1]	22.24 [16.48;30.01]	
***Subject household socio-demographic***					
**Household**		97.6%			0.076
Temporary/unplanned or slum	291 (9.1%)		62.2 [50.1;74.3]	Ref.	
Multi-floored building	277 (8.7%)		68.6 [59.0;78.0]	1.16 [0.79;1.71]	
Single-story attached building	1780 (55.7%)		69.0 [63.3;74.7]	1.25 [0.94;1.67]	
Single-story detached house	769 (24.1%)		72.4 [67.6;77.3]	1.47 [1.07;2.02]	
No data	77 (2.4%)		77.9 [69.5;86.3]	1.93 [1.04;3.59]	
**Living in the house since birth**		98.3%			0.547
Yes	2790 (87.3%)		69.7 [64.6;74.8]	Ref.	
No	351 (11.0%)		66.1 [58.4;73.7]	1.04 [0.81;1.35]	
No data	53 (1.7%)		75.5 [67.3;83.6]	1.47 [0.74;2.95]	
**Average monthly household income**		99.0%			0.320
<200000	910 (28.5%)		68.4 [63.0;76.7]	Ref.	
200,000–400,000	241 (7.5%)		68.0 [60.8;75.3]	0.86 [0.71;1.03]	
>400,000	2011 (63.0%)		69.9 [64.0;75.8]	1 [0.74;1.36]	
No data	32 (1%)		75 [65.8;84.2]	1.37 [0.60;3.16]	
**Parents/guardian highest education level**		99.4%			<0.0001
University	366 (11.5%)		60.4 [53.8;67.0]	Ref.	
Never went to formal school	92 (2.9%)		85.9 [79.0;92.8]	3.62 [1.87;7.00]	
Finished elementary school	628 (19.7%)		72.8 [64.8;80.7]	1.77 [1.30;2.40]	
Finished junior high school	707 (22.1%)		72.0 [65.8;78.2]	1.50 [1.12;2.00]	
Finished senior high school	1383 (43.3%)		67.7 [63.1;72.4]	1.18 [0.91;1.52]	
No data	18 (0.6%)		72.2 [56.8;87.6]	1.55 [0.52;4.60]	
**How many persons live in the household**		99.6%			<0.0001
1–3	491 (15.4%)		61.1 [54.4;67.8]	Ref.	
4–5	1803 (56.4%)		71.2 [66.4;76.1]	1.66 [1.33;2.07]	
>5	886 (27.7%)		70.2 [63.1;77.3]	1.52 [1.18;1.95]	
No data	14 (0.4%)		71.4 [50.4;72.4]	1.56 [0.47;5.19]	
***Dengue knowledge*, *exposure and control***					
**Heard about dengue before the study**		99.4%			0.168
No	236 (7.4%)		64.8 [52.0;77.6]	Ref.	
Yes	2940 (92.0%)		69.8 [65.0;74.5]	1.34 [0.99;1.82]	
No data	18 (0.6%)		66.7 [47.0;86.3]	1.29 [0.45;3.72]	
Knowledge of dengue illness symptoms*		91.7%			0.145
No symptoms known	9 (0.3%)		55.6 [11.5;99.6]	Ref.	
At least one symptoms	2919 (91.4%)		69.8 [65.1;74.5]	1.94 [0.47;7.97]	
No data	266 (8.3%)		65.4 [53.8;77.1]	1.50 [0.36;6.29]	
**How is dengue virus spreading among human**		91.1%			0.444
Mosquito bite	2573 (80.6%)		70.0 [65.6;74.4]	Ref.	
Other	336 (10.5%)		66.7 [57.1;76.3]	0.96 [0.72;1.28]	
No data	285 (8.9%)		67.0 [55.8;78.2]	0.83 [0.63;1.10]	
**When do mosquito bite**		86.0%			0.380
During the day	2603 (81.5%)		69.5 [65.1;73.3]	Ref.	
At night	145 (4.5%)		73.8 [62.1;85.5]	1.14 [0.76;1.69]	
No data	446 (14.0%)		67.3 [56.7;77.8]	0.87 [0.69;1.10]	
**Use insecticide spray to kill mosquitoes**		98.1%			0.903
No	2037 (63.8%)		68.6 [62.7;74.5]	Ref.	
Yes	110 (3.4%)		75.4 [57.3;93.6]	1.09 [0.67;1.78]	
Yes, all year long	733 (22.9%)		71.5 [67.3;75.6]	1.03 [0.84;1.27]	
Yes, during epidemics	254 (7.9%)		67.3 [60.2;74.4]	0.88 [0.65;1.20]	
No data	60 (1.9%)		68.3 [57.8;78.4]	0.92 [0.52;1.65]	
**Use mosquito mat/coil/liquid vaporizer**		97.8%			0.905
No	1385 (43.4%)		70.5 [64.8;76.2]	Ref.	
Yes	123 (3.8%)		71.5 [54.4;88.7]	1.04 [0.66;1.62]	
Yes, all year long	1210 (37.9%)		69.1 [64.2;74.0]	1.00 [0.83;1.21]	
Yes, during epidemics	406 (12.7%)		66.0 [58.2;73.8]	1.08 [0.83;1.40]	
No data	70 (2.2%)		67.1 [56.4;77.9]	0.81 [0.47;1.40]	
**Sleep under insecticidal bed net**		97.2%			0.555
No	2890 (90.5%)		70.1 [65.3;74.8]	Ref.	
Yes	23 (0.7%)		78.3 [60.0;96.6]	1.21 [0.42;3.44]	
Yes, all year long	179 (5.6%)		61.4 [49.1;73.8]	0.88 [0.62;1.24]	
Yes, during epidemics	13 (0.4%)		46.1 [10.5;81.8]	0.57 [1.18;1.81]	
No data	89 (2.8%)		65.2 [55.9;74.4]	0.74 [0.46;1.19]	
**Sleep under untreated bed net**		97.0%			0.102
No	2563 (80.2%)		70.7 [66.2;75.3]	Ref.	
Yes	43 (1.3%)		72.1 [57.2;86.9]	0.92 [0.46;1.86]	
Yes, all year long	454 (14.2%)		63.4 [52.2;74.7]	0.75 [0.59;0.95]	
Yes, during epidemics	39 (1.2%)		48.7 [32.9;64.5]	0.58 [0.30;1.13]	
No data	95 (3.0%)		69.5 [58.5;80.4]	0.86 [0.55;1.44]	
**Use air conditioner at home**		97.0%			0.168
No	2854 (89.3%)		69.8 [64.6;75.0]	Ref.	
Yes	20 (0.6%)		65.0 [36.6;93.4]	0.61 [0.23;1.62]	
Yes, all year long	208 (6.5%)		64.9 [55.1;74.7]	0.70 [0.51;0.96]	
Yes, during epidemics	16 (0.5%)		62.5 [38.8.86.2]	0.71 [0.25;2.05]	
No data	96 (3.0%)		68.7 [60.0;77.4]	0.82 [0.31;1.31]	
**Use mosquito repellent cream or spray**		97.5%			0.259
No	1714 (53.7%)		65.8 [59.7;71.9]	Ref.	
Yes	118 (3.7%)		72.0 [54.7;89.4]	0.90 [0.57;1.44]	
Yes, all year long	940 (29.4%		74.6 [70.2;78.9]	1.20 [0.98;1.47]	
Yes, during epidemics	341 (10.7%)		72.1 [66.2;78.0]	1.25 [0.95;1.66]	
No data	81 (2.5%)		69.1 [58.3;79.9]	1.01 [0.60;1.69]	
**Wear long clothing to protect from insect bite**		97.2%			0.791
No	2283 (71.5%)		69.3 [64.1;74.4]	Ref.	
Yes	112 (3.5%)		73.2 [55.5;91.0]	0.89 [0.56;1.43]	
Yes, all year long	521 (16.3%)		71.8 [64.2;79.4]	1.05 [0.83;1.33]	
Yes, during epidemics	187 (5.8%)		62.0 [51.9;72.1]	0.84 [0.59;1.19]	
No data	91 (2.8%)		69.2 [60.6;77.8]	0.91 [0.56;1.47]	
**Water container in the household**		99.3%			0.204
No	605 (18.9%)		72.1 [67.3;76.8]	Ref.	
Yes not covered	682 (21.3%)		67.7 [61.8;73.7]	0.94 [0.72;1.23]	
Yes tightly covered	1884 (59.0%)		69.3 [62.4;76.2]	0.94 [0.75;1.17]	
No data	23 (0.7%)		52.2 [34.9;69.4]	0.38 [0.16;0.92]	
**Check and eliminate stagnant water in the property**		97.3%			0.306
No	388 (12.1%)		72.4 [67.9;76.9]	Ref.	
Yes	329 (10.3%)		77.2 [65.7;88.7]	1.18 [0.81;1.73]	
Yes, all year long	2204 (69.0%)		68.4 [63.0;73.8]	0.86 [0.66;1.12]	
Yes, during epidemics	185 (5.8%)		64.9 [48.5;81.3]	0.87 [0.58;1.30]	
No data	88 (2.7%)		60.2 [54.2;66.2]	0.96 [0.55;1.67]	
***Dengue disease history***					
**Number of dengue cases in the household since the subject is born**		93.7%			<0.0001
No case	1500 (47.0%)		63.5 [56.2;70.7]	Ref.	
One case	288 (9.0%)		83.7 [78.7;88.7]	2.54 [1.80;3.60]	
>1 case	66 (2.1%)		87.9 [78.3;97.5]	3.83 [1.77;8.24]	
Don’t know	1140 (35.7%)		71.3 [66.2;76.4]	1.16 [0.93;1.46]	
No data	200 (6.3%)		76 [62.0;90.0]	1.27 [0.80;2.02]	
**Has a doctor ever diagnosed the subject with dengue**		92.9%			<0.0001
No	2406 (75.3%)		66.7 [61.4;72.1]	Ref.	
Yes	335 (10.5%)		81.5 [74.6;88.4]	2.27 [1.67;3.10]	
Don’t know	227 (7.1%)		69.2 [57.7;80.7]	1.05 [0.76;1.44]	
No data	226 (7.1%)		79.6 [70.0;89.3]	1.47 [0.95;2.28]	

*Dengue symptoms: bleeding, fever, headache, muscular pain, nausea and rash

In the final data set, the level of non-response (“no data”) varied from 0.4 to 14.0% (Table 1). Subjects were familiar with dengue disease, with 92% having heard about dengue and 91.4% able to cite at least one symptom. Control practices reported included use of repellent cream or mosquito spray (43.8%), elimination of mosquito breeding sites by covering water containers (59.0%) and eliminating stagnant water around the home (85.1%). Most subjects (75.3%) reported they had never been diagnosed with dengue.

Age and gender were associated with dengue serological status, with seroprevalences increasing with age (p<0.0001) and values of 71.1% (95% CI: 65.9–76.3) in females versus 67.4% (95% CI: 62.4–72.5) in males (p = 0.018) (Table 1). After univariate analysis, the type of household (p = 0.08), the level of education of the parents/guardians (p<0.0001), the number of persons living in the household (p<0.0001), knowledge about dengue symptoms (p = 0.14), sleeping under an untreated bed net (p = 0.10), the number of dengue cases identified since the subject was born (p<0.0001), and a previous clinical diagnosis of dengue for the subject (p<0.0001) were also associated with dengue serological status. In the multivariate model (Table 2), two variables remained associated with the dengue serologic status, the subject age group (1–4 vs 5–9 OR = 4.25; 1–4 vs. 10–14 OR = 12.60; and 1–4 vs 15–18 OR = 21.87; p<0.0001) and the number of cases diagnosed in the household since the subject was born (p = 0.0004).

**Table 2:** Result of the multivariate hierarchical logistic model of variables associated with dengue seropositive status.

Variable	N	Odd ratio [95% CI]	P-value
**Age group**			<0.0001
1–4	672 (21.0%)	Ref.	
5–9	861 (27.0%)	4.25 [3.39;5.37]	
10–14	886 (27.7%)	12.60 [9.72;16.35]	
15–18	775 (24.3%)	21.87 [16.16;29.59]	
**Number of dengue cases in the household since the subject is born**			0.0004
No	1500 (47.0%)	Ref.	
One	288 (9.0%)	2.05 [1.39;3.01]	
>1	66 (2.1%)	2.96 [1.29;6.79]	
Don’t know	1140 (35.7%)	1.06 [0.82;1.38]	
No data	200 (6.3%)	0.81 [0.48;1.36]	

### Force of infection

The constant force of infection model was valid and estimated a force of primary infection of 13.1% per year in dengue-naïve children. As a result of the goodness of fit statistic being close to 0.05, a model of varying force of infection (age groups of one year) was run to examine the homogeneity of the force of primary infection estimates per age group. As suggested by the first model, there was no clear trend in changes in force of infection with age; the estimates were overlapping, ranging from 10.2% to 18.5% per year. The highest force of primary infection was observed in the 1-year-old age group (Table 3).

**Table 3:** Dengue virus force of infection time varying and constant risk model.

Model		Estimated force of infection (%)	[95% CI]	P- value	Goodness of fit statistics
Model 1	1–18	13.1	12.5–13.6	<0.0001	>0.05**
Model 2	1	18.5	13.2–24.8	<0.0001	1.00***
	2	13.1	9.9–16.7	<0.0001	
	3	10.2	8.0–12.7	<0.0001	
	4	12.5	10.1–15.3	<0.0001	
	5	11.7	9.5–14.3	<0.0001	
	6	13.9	11.6–16.5	<0.0001	
	7	14.8	12.4–17.5	<0.0001	
	8	11.5	9.5–13.7	<0.0001	
	9	15.9	13.3–18.8	<0.0001	
	10	13.6	11.5–16.0	<0.0001	
	11	12.6	10.5–14.9	<0.0001	
	12	14.4	12.0–17.2	<0.0001	
	13	12.9	10.7–15.3	<0.0001	
	14	13.7	11.5–16.1	<0.0001	
	15	12.5	10.8–14.5	<0.0001	
	16	13.4	11.4–15.5	<0.0001	
	17	11.1	9.4–13.0	<0.0001	
	18	15.2	10.9–21.3	<0.0001	

** Pearson (0.063) and Deviance tests (0.068)

*** Hosmer and Lemeshow test

## Discussion

This is the first dengue antibody seroprevalence study conducted in a representative population of urban dwelling Indonesian children. The findings benefit from a cluster sampling design with probability proportional to size method, and sensitive and specific dengue diagnostic assays performed in the same laboratory.

This study found that 69.4% of children had been previously infected with dengue virus, more than 80% of children aged 10 years or over, indicating that the disease burden is extremely high. A seroprevalence study conducted in 1995 in healthy children in Yogyakarta, Indonesia, using the plaque reduction neutralization test to determine previous exposure, reported the presence of neutralizing antibodies in 56.2% of 4–9-year-old children, ranging from 37.2% in 4-year-old subjects to 69.7% in those 9 years of age. These are slightly lower than the rates observed in our study (Fig 3 and Table 1) and may be reflective of increasing dengue endemicity in the intervening decades, or geographic variability [19]. Our results also show higher levels of dengue virus exposure than those reported in other dengue endemic countries such as Sri Lanka (Colombo, 2008, 52.0% in those <12 years of age, and median age of seroconversion of 4.7 years) [13, 35], and Vietnam (Binh Thuan, 2003, 65.7% in 7–13 year olds) [14]. This elevated dengue exposure risk was also observed during a 2011 dengue vaccine trial in 5 Asian countries, where baseline dengue seroprevalence was highest in Indonesian children [36].

Our constant force of infection model estimated a 13.1% annual rate of primary infection among 1–18-year-old children, while the variable model estimated a force of infection that varied from 10.2% to 18.5%. These estimates are similar to those reported in Sri Lanka in 2008 (14.1% in those aged <12 years) and Southern Vietnam in 2003 (11.7% in 7–13-year-old children) [13, 14]. Despite these similarities between Vietnam and Indonesia in terms of transmission dynamics, the reported incidence of disease in Vietnam is more than twice that in Indonesia. [37]. A number of hypotheses could explain this difference in findings: most likely, it is reflective of Indonesia’s specific case definition for reported dengue disease (only DHF is reported), but underlying virological, genetic or epidemiological differences could play a role. From the constant force of primary infection model, it can be assumed that the average rate of primary infection was not highly variable over the past 18 years. Additional analysis may be needed to better understand infection risk over time. The recently observed increase in age distribution of reported cases may have been driven by more variable virologic, demographic, reporting or other determinants of disease [10]. A similar phenomenon was illustrated by a study conducted in Thailand showing that the upward shift in dengue case age was associated with demographic changes [38].

It can be assumed that dengue awareness, through social mobilization and education campaigns, begun in the 1970s, and the increasing public health importance associated with high media coverage, has steadily increased [39]. Knowledge of dengue transmission and symptoms was high within the study subjects; 92% of households had heard about dengue before our study and were able to cite at least one of the disease symptoms, and more than 80% knew that dengue virus is transmitted by diurnal mosquito bites. In term of exposure, household practices were focused on destroying mosquito breeding sites rather than personal protection. The level of exposure to the virus, however, is strong evidence that these reported behaviours are inadequate to protect against infection and additional prevention and control measures are urgently required.

In the multivariate model, only subject age group and the number of dengue cases that occurred in the household were associated with seropositive status. Some of the parameters significantly associated with dengue seropositivity in univariate models were also implicated in other dengue studies conducted in Latin America and Asia. For example, parental level of education and dengue illness history in the household have been associated with dengue seropositivity [17]. Other parameters, such as household size, exhibit an association inverse to that previously reported in the literature [40]. This is most likely explained by confounding effects from known risk factors such as age or unknown, socio-demographic drivers of exposure risk. The lack of significant associations between socio-demographic and behavioural factors with serological status provides evidence that essentially everyone is at risk of infection; that knowledge of prevention and control at the individual/household level is not protective against infection; and that additional measures to prevent transmission are required. The retrospective nature of our questionnaire limits the robustness of our results; recall bias may have been an issue.

A recent expansion in dengue virus transmission from urban to peri-urban and rural areas has been described [15] and the identification of provinces or areas of high transmission risk is a focus of prevention and control planning. This study showed a high level of exposure across urban Indonesia and, while we excluded rural areas from this study for operational reasons, it is likely that nearby peri-urban populations may have experienced similar high levels of exposure [40]. Another possible limitation is that cross-reaction between flaviviruses has been documented and the risk of false positives cannot be excluded. We consider this risk as low, because reports of other viruses such as Japanese encephalitis and Zika, in Indonesia, are rare. This study was not designed to make national-level infection or disease burden estimates but the observation that 13.1% of children suffer a primary infection per year translates into many millions of infections per year. Adults are presumably infected with a similar frequency. A proportion of these infections will be secondary, predisposing to symptomatic and severe disease. While a modelling approach would be required to quantify this burden, these data are strongly suggestive that dengue infections result in a significant burden of symptomatic and severe disease in urban Indonesia.

## Supporting information

S1 Checklist STROBE Checklist. (DOC)

S1 Appendix List of the 30 clusters selected in Indonesia. (DOCX)
