# Correction: Dengue seroprevalence and force of primary infection in a representative population of urban dwelling Indonesian children

**DOI:** 10.1371/journal.pntd.0006467

**Published:** 2018-05-02

**Authors:** Ari Prayitno, Anne-Frieda Taurel, Joshua Nealon, Hindra Irawan Satari, Mulya Rahma Karyanti, Rini Sekartini, Soedjatmiko Soedjatmiko, Hartono Gunardi, Bernie Endyarni Medise, R. Tedjo Sasmono, James Mark Simmerman, Alain Bouckenooghe, Sri Rezeki Hadinegoro

There are errors in Table 3 due to an incorrect calculation. The force of infection is *μ*, the mean number of primary infections per year or average rate at which susceptible individuals are infected, not *p*, the probability of a person living in the area being infected in one year. Please see the corrected Table 3 here.

**Table 3:** Dengue virus force of infection time varying and constant risk model.

Model		Estimate FOI (%)	[95% CI]	P- value	Goodness of fit statistics
Model 1	1–18	14.0	13.3–14.7	<0.0001	>0.05**
Model 2	1	20.5	14.2–28.5	<0.0001	1.00***
	2	14.0	10.4–18.3	<0.0001	
	3	10.8	8.3–13.6	<0.0001	
	4	13.4	10.7–16.6	<0.0001	
	5	12.5	10.0.15.4	<0.0001	
	6	15.0	12.3–18.1	<0.0001	
	7	16.0	13.2–19.2	<0.0001	
	8	12.2	10.0–14.7	<0.0001	
	9	17.3	14.3–20.9	<0.0001	
	10	14.7	12.2–17.4	<0.0001	

** Pearson (0.063) and Deviance tests (0.068)

*** Hosmer and Lemeshow test
