# Supplementary material for: Dengue seroprevalence and force of primary infection in a representative population of urban dwelling Indonesian children
Source: PLoS Negl Trop Dis. 2017 Jun 15;11(6):e0005621. doi: 10.1371/journal.pntd.0005621 (PMC5472274; doi:10.1371/journal.pntd.0005621)
Supplement: S1 Appendix — (DOCX) [file pntd.0005621.s002.docx]

S1 Appendix: List of the 30 clusters selected in Indonesia

| **Province** | **Regency/ Municipality** | **Subdistrict** |
| --- | --- | --- |
| NANGGROE ACEH DARUSSALAM | SUBULUSSALAM | SIMPANG KIRI |
| SUMATERA UTARA | MEDAN | MEDAN DENAI |
| SUMATERA BARAT | PADANG | PAUH |
| JAMBI | BUNGO | BUNGO DANI |
| LAMPUNG | LAMPUNG SELATAN | KALIANDA |
| BANTEN | TANGERANG | CIKUPA |
|  | TANGERANG | BENDA |
| DKI JAKARTA | JAKARTA SELATAN | PESANGGRAHAN |
|  | JAKARTA TIMUR | PULO GADUNG |
|  | JAKARTA BARAT | KALI DERES |
| JAWA BARAT | BOGOR | GUNUNG PUTRI |
|  | BANDUNG | BANJARAN |
|  | CIREBON | GUNUNG SARI |
|  | BEKASI | CIKARANG UTARA |
|  | BANDUNG | BOJONGLOA KALER |
|  | BEKASI | BEKASI TIMUR |
|  | TASIKMALAYA | SINGAPARNA |
| JAWA TENGAH | KLATEN | TRUCUK |
|  | JEPARA | PECANGAAN |
|  | TEGAL | DUKUHTURI |
|  | TEGAL | TEGAL BARAT |
| JAWA TIMUR | PONOROGO | PULUNG |
|  | BANYUWANGI | CLURING |
|  | MOJOKERTO | NGORO |
|  | SUMENEP | KALIANGET |
|  | SURABAYA | SAWAHAN |
| BALI | DENPASAR | DENPASAR SELATAN |
| KALIMANTAN TIMUR | SAMARINDA | SAMARINDA ULU |
| SULAWESI SELATAN | TORAJA UTARA | RANTEPAO |
| SULAWESI TENGGARA | KENDARI | KENDARI |
